# Platelets as central hubs of inflammation

**DOI:** 10.3389/fimmu.2025.1683553

**Published:** 2025-10-01

**Authors:** Yan Bo, Fei Zhao

**Affiliations:** ^1^ The Department of Medicine, Northwest Minzu University, Lanzhou, Gansu, China; ^2^ Key Laboratory of Environmental Ecology and Population Health in Northwest Minority Areas, Northwest Minzu University, Lanzhou, Gansu, China

**Keywords:** platelet immunology, inflammation, immune thrombosis, granule secretion, platelet–leukocyte interactions, pathogen recognition, tumor microenvironment, neuroinflammation

## Abstract

**Objective:**

To develop the platelet inflammation hypothesis and propose the concept of platelets as the central hub regulating inflammation.

**Methods:**

We employed a narrative review design. Based on platelets being the source of cellular fragments shed from megakaryocytes, we traced the active molecules within platelet granules to infer platelet regulatory roles in aseptic inflammation, infectious inflammation, cancer, and neuroinflammation. Furthermore, we visually mapped the central regulatory mechanisms of platelets in the aforementioned inflammatory contexts.

**Results:**

Platelets not only mediate hemostasis and thrombosis through the coagulation pathway but also dynamically regulate inflammatory responses through interactions between bioactive substances in platelet granules, leukocytes, vasculature, and immune signaling. This regulatory role applies across a broad spectrum of pathological inflammations. Platelets influence vascular integrity in aseptic inflammatory injury, participate in pathogen recognition and containment during infectious inflammation, and regulate immune cell recruitment and inflammatory outcomes in tumor/cancer and neuroinflammation. The central principle of platelet-mediated inflammation regulation is dual control of immune thrombogenesis through damage-associated molecular patterns (DAMPs) and pathogen-associated molecular patterns (PAMPs), thereby influencing disease outcomes.

**Conclusion:**

Platelets serve as the central hub in the microcirculatory-damaged tissue-immune inflammatory interaction network. Their immunoregulatory functions play a pivotal role in diverse inflammatory pathologies. The platelet-driven mechanism-disease-immune inflammatory regulation framework provides clinically translatable insights for diagnostic evaluation of inflammatory and thrombotic conditions, as well as for developing antiplatelet therapeutic strategies targeting diseases such as cancer and epilepsy.

## 1 Introduction

Inflammation is a fundamental pathological response triggered by physical, chemical, or pathogen-induced damage to cells, tissues, or organs. The inflammatory response involves complex interactions among vascular, immune, and extracellular matrix components, ultimately aiming to stabilize the physiological properties of tissue cells (1). Historically, platelets were regarded as hemostatic sentinels capable of rapidly aggregating at sites of vascular rupture (2). Over the past four decades, mounting evidence has suggested a redefinition of platelet biological functions. As cellular fragments shed from megakaryocytes, platelets possess the potential to act as regulators of immune function, with the capacity to modulate both innate and adaptive immunity (3, 4).

The cytoplasm of platelets contains abundant bioactive substances, broadly categorized into α-granules, dense granules, and lysosomes, which recognize, release, and modulate inflammatory signals (5–7). The contents of these platelet granules are fundamental to platelet function, directly participating in physical and biochemical interactions with leukocytes and secreting mediators that amplify the inflammatory cascade (8, 9). This platelet-associated inflammation occurs across diverse pathological contexts. Examples include aseptic inflammation linked to damage-associated molecular patterns (DAMPs) (4, 10); antimicrobial defense against bacterial, fungal, viral, and parasitic pathogens associated with pathogen-associated molecular patterns (PAMPs) (3, 11–15); tumor-associated inflammation (16–18); and neuroimmune inflammation exemplified by epilepsy (19–21).

Although human understanding of platelets is gradually deepening, the concept of integrating hemostatic and immune functions into platelets’ central role in inflammation has yet to be proposed. The purpose of this review is to examine the molecular and cellular pathways through which platelets regulate inflammatory processes and to establish a framework applicable across different disease contexts. This may unlock the potential for platelets to serve as therapeutic targets and diagnostic biomarkers. From this perspective, platelets function not merely as auxiliary participants in immunity but as key regulators whose influence extends from maintaining vascular integrity to coordinating systemic immune surveillance. Based on this consideration, platelets hold promise as a low-cost component for clinically assessing systemic inflammation and immunity.

## 2 Methods

In the Lanzhou region of China, clinicians typically assess patients’ inflammatory states by evaluating white blood cell counts and changes in the ratios of different white blood cell subtypes in blood samples. Platelets originate from fragments containing cytoplasm shed by megakaryocytes. Based on this platelet origin, Dr. Yan Bo proposed a platelet inflammation hypothesis in March 2022 (22) and publicly disseminated it at the 2nd International Conference on Biological Engineering and Medical Science (23). Platelets serve as a central hub for communicating with inflammatory cells and cytokines, thereby promoting the onset, progression, and resolution of inflammation. The preliminary clinical significance of the platelet inflammation hypothesis lies in providing clinicians in regions with unequal medical resources and economic hardship with an additional perspective for assessing inflammatory states.

To further develop the platelet inflammation hypothesis proposed by Dr. Yan Bo, this study adopted a narrative review design. The literature retrieval process relied on the previously proposed hypothesis (23) and the methodologies employed in Dr. Yan Bo’s prior research (24–26). The specific formula for executing the literature search referenced the studies by Fei Zhao et al. (27–29). We conducted a qualitative synthesis of evidence from two perspectives: platelet granule biology and pathophysiologic contexts. To narrow the scope of evidence synthesis, platelet granule biology described α-granules, dense granules, and lysosomes, while pathophysiologic contexts selected sterile injury/immunothrombosis, infection, tumor microenvironment, and neuroinflammation/epilepsy. We attempted to visualize this platelet inflammation hypothesis.

## 3 Platelet biology in the context of inflammation

### 3.1 Granule composition and functional molecules

**Figure 1:**
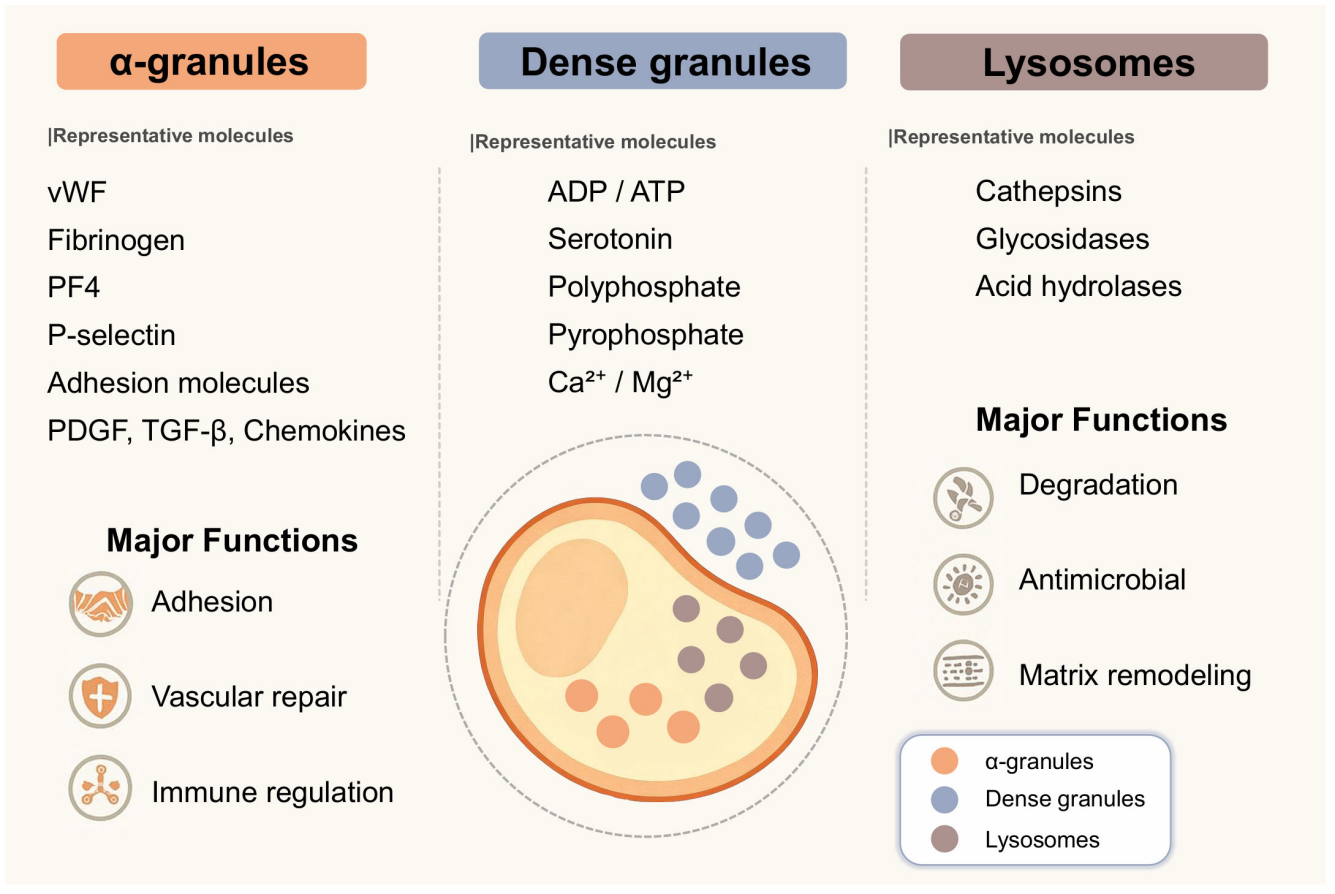
Schematic diagram of platelet morphology. This illustration by Figdraw primarily depicts the types of platelet granules within the cytoplasm. True platelets exhibit a biconcave disc shape. The diagram shows the cytoplasm (yellow region), the core area where cytoplasm concentrates (orange ellipse), dense granules (small blue dots), α-granules (small orange dots), and lysosomes (small gray dots). The image lists the primary molecular representatives for each platelet granule type and provides a highly generalized description of their functions. α-granules contain adhesion molecules such as P-selectin and GPIIb/IIIa, growth factors including PDGF and VEGF, chemokines such as CXCL4 and CCL5, and complement components. Dense granules store small molecules, including ADP, ATP, serotonin, polyphosphate (polyP), and divalent cations such as calcium and magnesium. Lysosomes contain degradative enzymes such as cathepsin D, glycosidases, and acid hydrolases that participate in extracellular matrix remodeling and antimicrobial activity. In fact, each unique molecule plays a distinct role in platelets’ regulation of immune function.

The pro-inflammatory and immune functions of platelets, in addition to their aggregation capabilities, originate from stored secretory granules. These granules contain a rich array of bioactive molecules. Previous researchers have classified secretory granules into α-granules, dense granules, and lysosomes. Each granule type harbors numerous unique molecules. The spatial organization of these granules within platelets and their distinct release signaling mechanisms collectively regulate immune and inflammatory responses in vascular and interstitial spaces. We employ a visual approach to summarize platelet secretory granule types, their representative molecules, and primary functions, providing a molecular functional reference for subsequent discussion (**Figure 1**). We then investigate how these platelet granules operate during aseptic inflammatory injury and immune thrombosis.

Platelets primarily consist of three types of secretory granules: α-granules, dense granules, and lysosomes (5). Each secretory granule contains specific bioactive molecules that work synergistically to mediate platelet functions in hemostasis and repair, immune regulation, and inflammatory signaling.

Alpha granules contain the highest number of active molecules. Among these, molecules that mediate adhesion are collectively termed adhesion molecules, such as P-selectin, platelet-endothelial cell adhesion molecule-1 (PECAM-1), and GPIIb/IIIa. These adhesion molecules facilitate interactions between platelets, leukocytes, and endothelial cells, thereby promoting immune cell recruitment (5–7). α-granules also contain hemostatic proteins and angiogenic mediators supporting hemostasis and repair functions. Hemostatic proteins include von Willebrand factor (vWF) and fibrinogen, while angiogenic mediators comprise vascular endothelial growth factor (VEGF), angiopoietin, and anti-angiogenic factors. Typically, anti-angiogenic factors in platelets primarily refer to platelet factor 4 (PF4). Certain growth factors that are difficult to categorize, such as platelet-derived growth factor (PDGF), basic fibroblast growth factor (bFGF), and stromal cell–derived factor 1α (SDF-1α), contribute to tissue regeneration and leukocyte chemotaxis (5, 6). Immunomodulatory chemokines such as CXCL4, CCL5, and CXCL7 derivatives coordinate the activation and migration of neutrophils, monocytes, and lymphocytes. Complement proteins like C3 and C3b, along with their regulatory factors, enhance the antimicrobial defense functions of the innate immune barrier (7).

Dense granules are enriched in small molecules, including adenosine diphosphate (ADP), serotonin, polyphosphate, pyrophosphate, calcium, and magnesium ions (8, 9). These compounds intensify platelet aggregation, influence vascular tone, and act as paracrine signals to modulate leukocyte activation and endothelial permeability. Polyphosphate has also been linked to the initiation of the contact pathway of coagulation and the stabilization of immune thrombi.

Lysosomes contain degradative enzymes, including cathepsin D, glycosidases, and other acid hydrolases (30). Associated with intracellular digestion, these enzymes can also be secreted into the extracellular space, where they degrade microbial cell walls and remodel damaged extracellular matrix, shaping the inflammatory microenvironment.

### 3.2 Platelet–leukocyte crosstalk and immune thrombosis

The interaction between platelets and leukocytes primarily occurs through two pathways: receptor-ligand interactions and the release of soluble mediators. Neutrophils, acting as sentinel immune cells, participate in acute immune responses, and the formation of immune thrombi through platelet-neutrophil adhesion is crucial to this process.

The synergistic mechanism between the platelet coagulation pathway and innate immunity underlies the principle of immune thrombosis. Immune thrombosis can function as a local inflammatory barrier to limit pathogen spread by impairing the thrombotic inflammatory barrier. Following endothelial injury recognition, platelets adhere to fibrin-deposited structural scaffolds and progressively aggregate into immune thrombi. This specialized fibrin scaffold is believed to be composed of neutrophil extracellular traps (NETs), comprising chromatin fibers and granular proteins (31–34). Platelet adhesion to NETs is guided by P-selectin, which first binds to PSGL-1 on neutrophils, subsequently activating the scaffold structure. Subsequently, platelet-derived chemokines and polyphosphates further promote neutrophil recruitment and NETs activation.

Although platelet-driven immune thrombosis may exert a protective role during acute pathogen infection by limiting pathogen spread, dysregulated activation guided by platelet reserves can trigger microvascular occlusion, tissue ischemia, and amplified inflammation. This dual nature underscores the critical importance of platelets and neutrophils in maintaining a delicate balance between dynamic host defense and preserving tissue integrity.

In this summary, we outline the process by which platelets and leukocytes synergistically form immune-mediated thrombi. This process is initiated by the activation of endothelial cells following inflammatory or infectious stimuli, with such injury promoting leukocyte adhesion and platelet recruitment. Functionally activated platelets release stored granule mediators within their cytoplasm, which can promote leukocyte activation. During this process, leukocytes, particularly neutrophils, form NETs, ingeniously trapping platelets and pathogens. The interaction between platelets and leukocytes facilitates pathogen containment while maintaining the fundamental immune function of occluding blood vessels in pathological injury.

**Figure 2:**
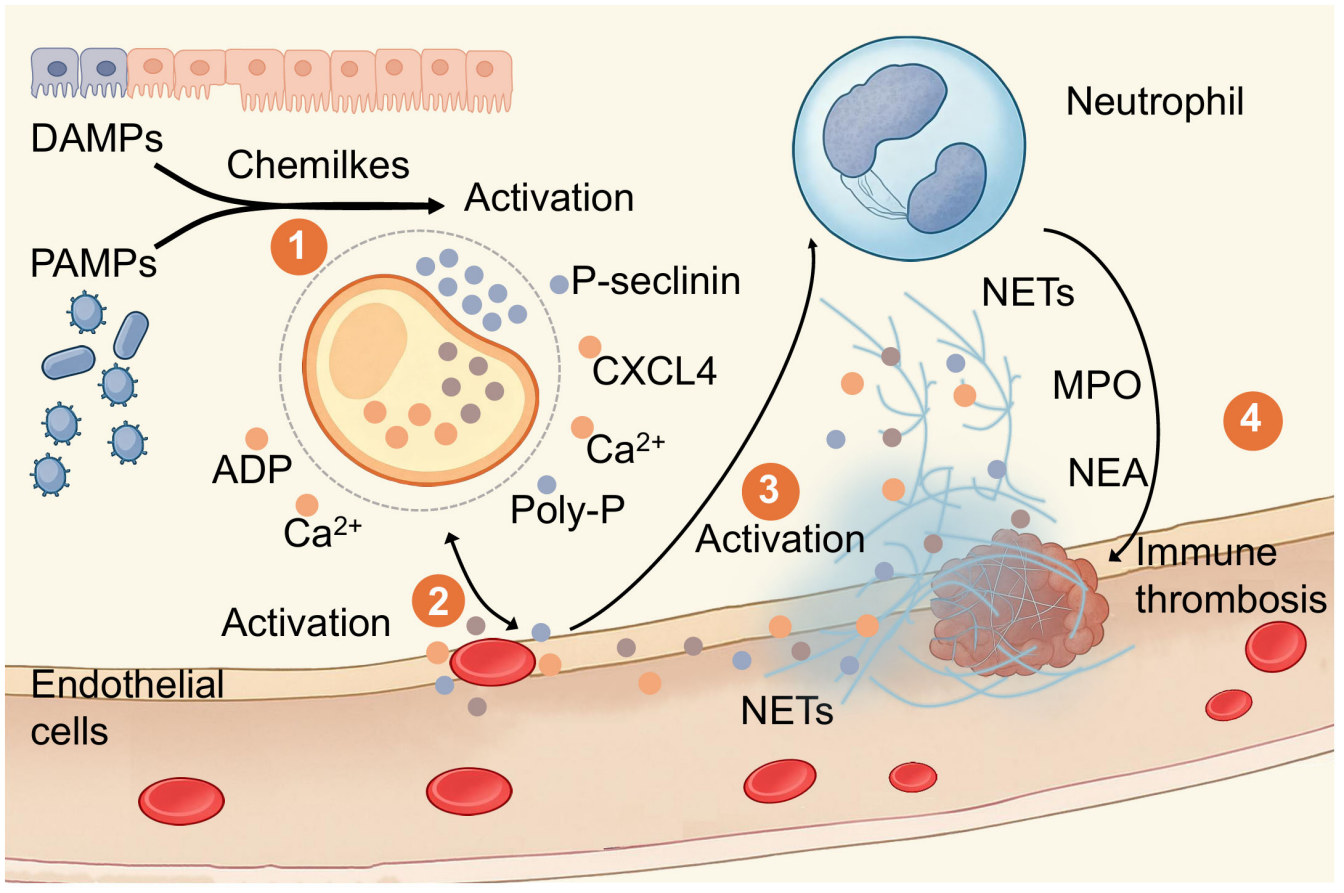
Overview of the Mechanism of Immune Thrombosis. Illustration by Figdraw depicting the activation of immune responses and thrombus formation. damage-associated molecular patterns (DAMPs) and pathogen-associated molecular patterns (PAMPs) lead to activation, releasing ADP and calcium ions. Endothelial cells activate, followed by a neutrophil, releasing NETs, myeloperoxidase (MPO), and neutrophil elastase (NEA). This process contributes to immune thrombosis, highlighted in four labeled steps. Step 1 involves damaged endothelial cells activating platelets. Platelets recognize damaged endothelial cells via two pathways leading to ICAM-1 expression: DAMPs and PAMPs. Step 2 involves platelet adhesion to damaged endothelial cells. Platelets release adhesion molecules such as P-selectin, intercellular adhesion molecule-1 (ICAM-1), and polyphosphate (polyP), facilitating migration and adhesion to the surface of damaged endothelial cells. During platelet adhesion, continued release occurs from platelet α-granules and dense granules, including CXCL4, ADP, and calcium ions. Step 3 involves platelet activation and NET formation. Neutrophils interact with platelet-released P-selectin and PSGL-1, along with MPO and NEA, progressively forming neutrophil extracellular traps. Step 4 involves the formation and effector functions of immune thrombi. Fibrin and platelet adhesion and aggregation enhance NETs' ability to capture pathogens or damaged endothelial cells, forming immune thrombi. This process restricts local microcirculatory blood flow while simultaneously exerting innate immune defense functions. This represents an integrated perspective of coagulation and immune defense.

We summarized the cellular and molecular interactions underlying immune thrombogenesis, integrating steps from initial activation to thrombus formation (**Figure 2**). Building on this foundation, we shifted focus to pathogen-driven environments, where platelet-NETs consistently exerted outcome-oriented effects across diverse pathogen-driven patterns, thereby bolstering the credibility of the platelet inflammation hypothesis.

## 4 Platelets in different inflammatory contexts

### 4.1 Sterile inflammation

Aseptic inflammation is an inflammatory response triggered by the release of endogenous signals during non-pathogen–induced cellular injury or stress processes. This process is more covert compared to pathogen-induced infection-related inflammation. However, there are similarities between aseptic inflammation and infection-related inflammation in terms of innate immune cell recruitment and signaling pathway activation. The distinction lies in the fact that aseptic inflammation is triggered by different mediators of damage-associated molecular patterns (DAMPs) (4, 10).

Atherosclerosis represents the most characteristic non-infectious inflammatory disease. From a pathological perspective, activated platelets play a decisive role in the onset and progression of atherosclerotic disease (4). Furthermore, gout is a clinical condition characterized by non-infectious inflammatory manifestations arising from an autoimmune inflammatory disorder. This is associated with platelet count and morphology (4). During tissue injury in such aseptic inflammation, damaged endothelial cells release interleukin-1α, S100 proteins, and heat shock proteins (HSPs) into the microcirculation. These molecules directly activate monocytes, macrophages, and dendritic cells via the DAMPs pathway. These activated immune cells, in turn, release inflammatory mediators against the concentration gradient, recruiting additional immune cells to promote platelet binding (10, 35).

Platelets can respond to the DAMPs pathway because the α-granules and dense granules released by platelets contain chemokines, adhesion molecules, and procoagulant factors. These are precisely the inflammatory mediators that recruit leukocytes against a chemical gradient. This not only promotes NET formation but also further amplifies inflammatory signaling (36). Evidence from inflammatory arthritis models suggests that assessing platelet function via patient venous blood samples may guide therapeutic strategies for inflammatory arthritis (37). Interestingly, platelet activity contributes to both immunoregulation and tissue repair, highlighting their dual capacity to promote and resolve inflammation (38).

Multiple clinical observations have identified correlations between platelet indices and sterile inflammatory diseases. Elevated neutrophil-to-lymphocyte ratios (NLR) and platelet-to-lymphocyte ratios (PLR) have been associated with microvascular inflammation in proliferative diabetic retinopathy (39) and systemic inflammatory activity in familial Mediterranean fever, although PLR may be less predictive in certain contexts (40). Another study suggests a tripartite relationship among neutrophils, platelets, and systemic inflammation: cathepsin G and elastase released by activated neutrophils can activate platelets via adenosine diphosphate-dependent pathways, thereby inducing inflammatory responses (41).

These findings suggest that platelet activation may constitute a key component of aseptic inflammation. However, damaged tissues release distinct molecular signals that induce platelets to exert a dual role in modulating the inflammatory microenvironment. In other words, platelets both contribute to enhancing host defense and promote the development of non-infectious inflammatory diseases.

### 4.2 Pathogen-driven inflammation

**Figure 3:**
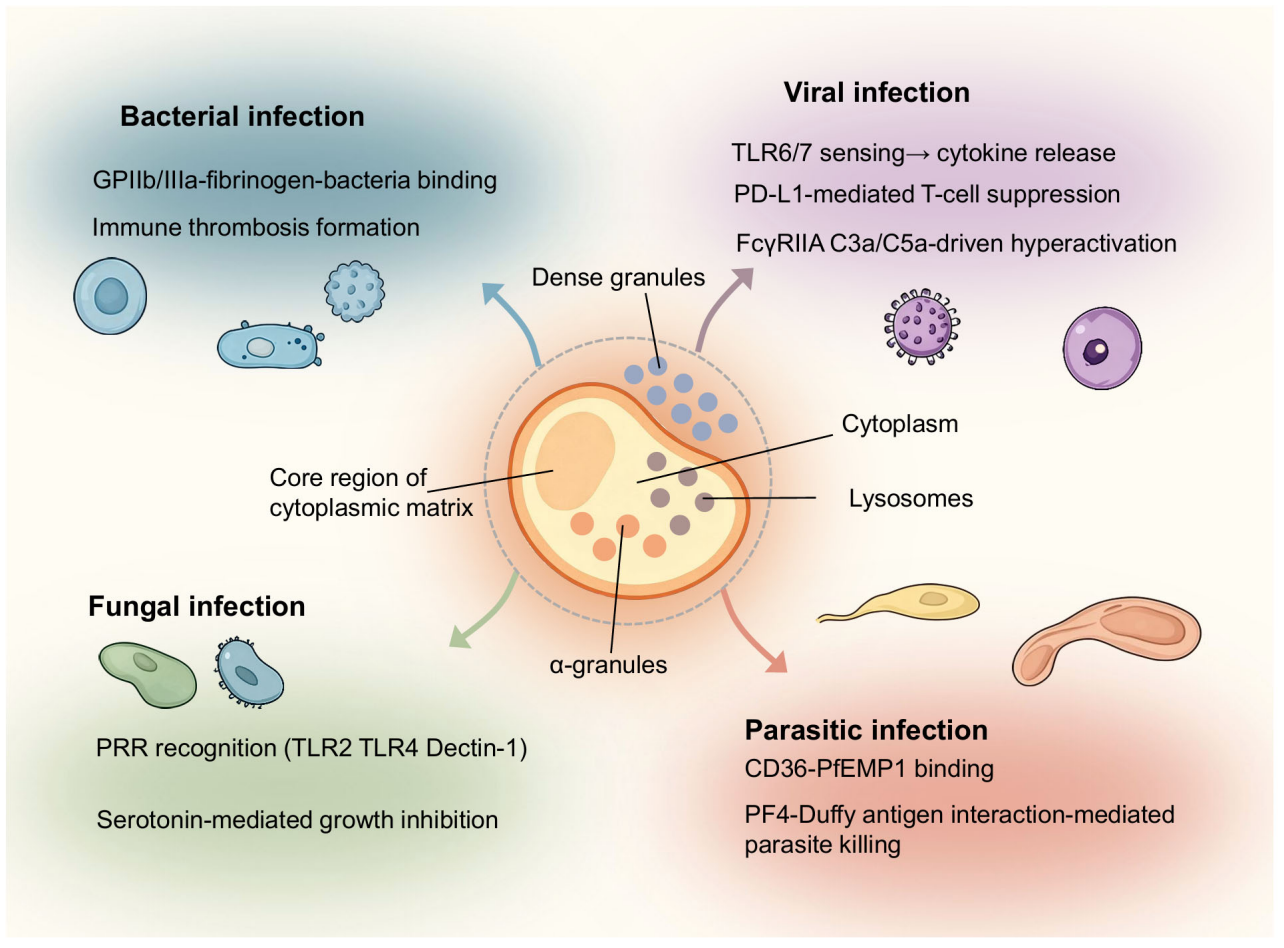
Overview of platelet regulatory mechanisms in infections caused by different classes of pathogens. The schematic positions a central platelet hub in relation to four categories of infectious agents: bacteria, fungi, viruses, and parasites. In bacterial infections, platelets bind pathogens via GPIIb/IIIa and fibrinogen, release PF4 and RANTES, and contribute to immune thrombosis. In fungal infections, pattern recognition receptors such as TLR2, TLR4, and Dectin-1 mediate platelet activation, leading to serotonin release and β-glucan–dependent immune responses. Viral infections engage pathways including TLR7 and TLR8 sensing, PD-L1–mediated T-cell modulation, and FcγRIIA and complement C5a–driven hyperactivation. Parasitic infections, exemplified by Plasmodium species, involve CD36 and PfEMP1 binding as well as PF4 interaction with the Duffy antigen to promote parasite clearance. The diagram by Figdraw emphasizes the diversity of platelet functions while underscoring shared roles in pathogen recognition, immune amplification, and thrombotic regulation.

Inflammation caused by pathogens typically commands patient attention, such as sudden fever symptoms. Compared to other inflammatory contexts, we expand our description of pathogen-induced inflammation by classifying it according to bacterial, fungal, viral, and parasitic origins. Once pathogens invade the body and trigger localized inflammation, platelets recognize the pathogens through inflammatory signals and respond by migrating, aggregating, and forming immune-mediated thrombi. This process activates the antimicrobial defense functions of the classical complement pathway. Inflammatory signaling molecules within platelet granules can suppress pathogens, recruit immune cells, and modulate the inflammatory microenvironment through receptor-mediated signaling pathways. Platelet responses to infection vary depending on pathogen type, highlighting not only differences in microbial structure and host receptor specificity but also the highly adaptive nature of platelet-guided inflammation. Although molecular signaling and cell-cell interactions differ slightly among bacteria, fungi, viruses, and parasites, platelets have progressively evolved universal principles of response. By recognizing pathogen-associated molecular patterns, platelets release a cascade of granules that resist pathogens and promote inflammation. This not only enhances platelet-leukocyte interactions but also amplifies the immune-inflammatory response. We attempt to summarize this platelet-regulated mechanism governing pathogen-driven inflammation. We highlight both conserved pathways and pathogen-specific strategies through which platelets influence infectious disease progression (**Figure 3**). Subsequently, we explore the unique inflammatory contexts of tumors and the brain, which may spark public discussion and concern.

#### 4.2.1 Bacterial infections

Platelets defend against bacterial infections through adhesion, aggregation, and triggering inflammatory signals. Typically, platelets form an immune thrombus resembling a cage, confining the bacteria. Subsequently, platelets initiate repair functions, whereby the immune thrombus and the confined bacteria undergo encapsulation, calcification, phagocytosis, and absorption as a unified entity (11). *Streptococcus pyogenes* can be captured when fibrinogen-bound platelet GPIIb/IIIa receptors transmit activation signals, leading to platelet degranulation and the release of inflammatory mediators (3). These mediators include regulated upon activation normal T-cell expressed and secreted (RANTES), platelet factor 4 (PF4), soluble CD40 ligand, soluble P-selectin, platelet-derived growth factor (PDGF), and adenosine diphosphate (ADP), all of which can enhance leukocyte recruitment and activation (42, 43). Such coordinated responses integrate coagulation with innate immunity, providing both a physical barrier and biochemical antimicrobial activity.

#### 4.2.2 Fungal infections

Platelets can recognize fungal components through pattern recognition receptors by activating protease-activated receptors (PARs) and toll-like receptors (TLRs) (12, 13, 44). Upon detecting polysaccharide structures such as β-1,3-glucan and β-galactomannan, these receptors initiate signaling cascades to exert antifungal biological functions. Certain glucans also confer protection against Aspergillus infection, possibly by enhancing host immune responses (45). Antimicrobial molecules released from platelet granules, such as serotonin, exhibit direct inhibitory effects on fungal growth *in vitro* (46). From this perspective, platelets function more as enhancers that modulate the fungal-driven inflammatory process by assisting neutrophils and macrophages.

#### 4.2.3 Viral infections

Platelets can recognize and internalize viral particles to promote immune responses against viral infection, with the recognition and internalization processes requiring activation of TLR-dependent pathways (9). An HIV study demonstrated that platelets in patient blood samples undergoing antiretroviral therapy contained replicating viruses detectable by electron microscopy. This confirms that platelets possess the capacity to phagocytose viruses, specifically demonstrated with HIV. Of course, after phagocytosing HIV, platelets may provide shelter rather than immune killing (47). Clinical observations of influenza virus infection suggest this perspective. In patients with severe influenza virus infection, platelet counts gradually decline, indicating that platelet consumption translates into an additional immune response (14). In COVID-19 research, platelets have been shown to regulate CD4^+^ T-cell function via PD-L1-dependent mechanisms (48). During SARS-CoV-2 infection, platelets primarily respond to the inflammatory response induced by the spike protein by activating monocytes (49) and activating the complement pathway (50). Platelet-mediated promotion of the COVID-19 immune response via the complement pathway may be associated with hyperactivation driven by FcγRIIA and complement C5a signaling (50). This evidence indicates that platelets function as both immune effectors and immune enhancers in viral infectious diseases.

#### 4.2.4 Parasitic infections

The host’s immune response to most parasites is categorized as parasitogenic immunity. This means that when parasites invade the host, immune cells can mount an immune response and convert it into stored immune memory. If the parasites are eliminated during the immune response, the previously converted immune memory will disappear, failing to provide protection against reinfection. This characteristic of parasitogenic immunity hinders immunological research on parasitic infections. A parasitic infection closely associated with platelets is malaria, which invades red blood cells. Platelets can target, recognize, and bind infected red blood cells via the scavenger receptor CD36 and *Plasmodium falciparum* erythrocyte membrane protein 1 (PfEMP1) (51, 52). From an antigen exposure perspective, this effectively transfers the parasite’s antigenic proteins to the red blood cell surface. This mechanism may suppress the parasite’s immune evasion strategy. Platelet factor 4 binds to the Duffy antigen on red blood cells, representing a critical step in the immune response to kill the parasite (15, 53). These mechanisms highlight the evolutionary adaptation of platelets in anti-parasitic immunity, acting in concert with both innate and adaptive immune pathways.

Against the backdrop of fungal, bacterial, viral, and parasitic infections we have enumerated above, the common functional modules exhibited by platelet-driven immunity include recognizing pathogen-associated molecular patterns, local hemostasis, activating inflammatory pathways, and delivering immune effectors to the site of infection. This phenomenon underscores the central role of platelets in regulating inflammation during pathogen-driven inflammatory states.

### 4.3 Tumor-associated inflammation

In the tumor microenvironment, the effector role of platelets in inflammation is diametrically opposed to the previously mentioned aseptic inflammation and pathogen-driven inflammation. Activated platelets can adhere to tumor cell surfaces, forming microthrombi that shield tumor cells from circulatory shear stress and immune cell recognition (16). This platelet-mediated immune evasion enhances tumor cell survival during migration and dissemination.

Platelet-associated tumor adhesion molecules involve multiple ligand-receptor systems. Podoplanin (PDPN) is a sialylated membrane glycoprotein expressed in various common tumor types. PDPN binds to platelet C-type lectin receptor 2 (CLEC-2), triggering platelet activation and aggregation (18). Interactions between platelets and tumor cells frequently accompany tumor metastasis. In experimental models, this platelet action promotes venous thrombosis in ovarian cancer. Ovarian cancer cells can internalize platelets via dynein-dependent endocytosis, allowing metabolic substrates and signaling molecules contained within platelet granules to support ovarian cancer cell growth (17).

Platelet-mediated tumor immune escape is not entirely without research value. Existing studies suggest that low molecular weight protein tyrosine phosphatase (LMWPTP) can interfere with tumor-platelet interactions. If LMWPTP expression is upregulated in tumor cells, their proliferation capacity, metastatic potential, and chemotherapy resistance are significantly enhanced. This phenomenon was first proposed by Chiarugi et al. in 1995, who suggested LMWPTP catalyzes the phosphorylation of platelet-derived growth factor receptors (PDGFR) (54). Two experimental studies in 1998 demonstrated that LMWPTP influences the duration of tyrosine phosphorylation and alters fos proto-oncogene expression by enhancing PDGF catalytic activity (55, 56). As an enzyme involved in early mitotic events, LMWPTP also regulates the phosphorylation state of p190Rho-GAP to control cytoskeletal reorganization, subsequently counter-regulating PDGF (57). Why does LMWPTP upregulation enhance tumor proliferation and migration? Because LMWPTP strongly influences PDGF-stimulated cell adhesion, spreading, and chemotaxis by regulating p190Rho-GAP phosphorylation levels. This establishes the foundation for tumor cell migration. Meanwhile, phosphorylation at tyrosine 131 affects mitotic progression, dephosphorylation of activated PDGF-R, and cytoskeletal reorganization, acting on p190RhoGAP. This establishes the foundation for tumor cell proliferation (58). A crucial fact to recognize is that the proliferation process of tumor cells can further increase LMWPTP expression, thereby creating a positive feedback loop in tumor development. As of 2019, the concept of antiplatelet therapy for tumors remained focused on overcoming tumor-associated thromboembolism (59).

In addition to promoting tumor cell metastasis, platelets can also regulate inflammation during tumor cell-mediated matrix remodeling. This process shares similarities with angiogenesis, relying on growth factors, cytokines, and proangiogenic mediators within platelet granules. In current clinical practice, the inflammatory dynamics between platelets and tumors have led to the development of a composite inflammatory prediction method capable of assessing the Systemic Inflammatory Response Index (SIRI). Currently, SIRI is applied in clinical practice as a prognostic indicator for breast and gastric cancers (60). The primary clinical significance of this discovery lies in enabling clinicians to use specific mathematical models to fit platelet parameters, thereby intuitively assessing tumor inflammation and drug resistance levels.

The aforementioned relationship between platelets and tumor inflammation highlights a potential perspective: inhibiting platelet activity represents a novel therapeutic approach for cancer patients. Here, we take aspirin, a representative anticoagulant, as an example. Inhibiting activation or adhesion with aspirin, P2Y12 blockade, antagonism of the P-selectin/PSGL-1 interface, or experimental interference with the CLEC-2/podoplanin axis may weaken platelet cloaking of tumor cells, dampen tumor-associated immunothrombosis, and constrain pro-angiogenic signaling. This approach carries a risk of bleeding, a clinical risk that is heightened in the perioperative period, in cases of mucosal invasion, and in chemotherapy-induced thrombocytopenia. Naturally, the host defense function involving the synergistic action of platelets and leukocytes is also diminished. Clinical decision making should weigh the net effect of aspirin-induced platelet inhibition on the patient. We recommend that the potential value of anti-tumor inflammatory, and anti-thrombotic effects should at least outweigh the risks of bleeding and compromised host immune function.

Based on anti-tumor inflammatory treatment strategies that suppress platelet activity, we propose several treatment timing options for consideration. During the surgical phase of tumor resection and periods with low infection risk, we recommend short-term pulse therapy at the lowest dose. During pulse therapy, we suggest using platelet-related biomarkers to assess the progression of anti-tumor inflammatory therapy that suppresses platelet activity. Examples include platelet-leukocyte aggregates, soluble P-selectin, the ratio of CXCL4 to PF4 concentrations, and SIRI. Furthermore, we suggest integrating the immune-inflammatory thrombosis state described in our platelet inflammation hypothesis with tumor metastasis-related biomarkers to enhance clinical decision-making utility. Once the therapeutic window for tumor treatment can be relatively objectively characterized from multiple perspectives, clinicians will gain greater confidence in balancing the benefits and risks of antiplatelet therapy for tumors.

From the perspective of tumor-driven inflammation, platelets enhance mechanisms of immune evasion by tumor cells while simultaneously increasing the tolerance of physical barriers to tumor cell defense. This unique negative feedback effect enables us to propose an intriguing therapeutic strategy: inhibiting platelet-tumor interactions to suppress tumor inflammatory processes. Finally, we extend this analogous mechanism to neuroinflammation.

### 4.4 Platelet-driven neuroinflammation in epilepsy

The central nervous system (CNS) has long been considered an immune-privileged region. This widely accepted notion stems from the blood–brain barrier (BBB), which restricts the entry of circulating immune cells and molecules into the CNS (19). However, under the pathological conditions of epileptic seizures, the BBB, locally damaged by abnormal discharges, loses its selective permeability, allowing platelets to participate in neuroinflammation. This outcome disrupts the CNS’s inherent immune homeostasis.

During an epileptic seizure, the physical convulsions experienced by patients result from abnormal electrical discharges in the brain’s neurons. In such events, sustained abnormal neuronal currents may compromise the structural integrity of the BBB by damaging the basement membrane or cell membranes of the endothelial cells that form it. This disruption of the BBB structure creates a breach in the barrier, allowing plasma proteins and other blood-derived substances to leak into the barrier’s interior. Substances circulating in the peripheral circulation may become immunogenic during CNS circulation, causing persistent immune damage to blood vessels or neurons. Activated platelets are recruited to sites of vascular injury and promote BBB repair. This process is guided by Toll-like receptors (TLRs) on immune cells (20). As discussed in the aseptic inflammation section, platelets attract immune effector cells during inflammation, particularly astrocytes, microglia, and mast cells within the CNS.

Here it is necessary to describe the shift in concepts regarding epilepsy treatment, which aids the reliability of our reasoning. In the PubMed database, the earliest study tracing the relationship between epilepsy and platelets we could find was a clinical study reported by Airaksinen. In the trial, the levels at which platelets from epilepsy patients could take up four neurotransmitters—taurine, GABA, 5-HT, and dopamine—were approximately 70%–80% of those in healthy individuals (61). Initially, we hypothesized that seizures or antiepileptic drug use might deplete reserve granules in platelets, leading to reduced platelet activity and impaired uptake. However, another clinical observation revealed higher monoamine oxidase levels in platelets of epilepsy patients compared to healthy individuals, refuting the platelet depletion hypothesis (62). This was resolved when we discovered that both the brain and platelets contain two isoforms of human phenol sulfotransferase: a heat-resistant type and a heat-labile type. If epileptic abnormal discharges generate additional electrical heat, the heat-resistant isoform becomes predominant. This enzyme’s biological function is to catalyze the sulfation coupling of dopamine and other monoamines. However, in epileptic patients, platelet-associated thermostable HPST exhibits a high correlation with the thermostable subtype in the brain (63). Early clinical epilepsy studies treated seizures by inhibiting GABAase activity in platelets from a neuroexcitatory perspective (64). An early clinical study measuring taurine in platelets confirmed the concept of platelet-related neural excitation (65). Subsequently, some patients treated with valproate for epilepsy developed thrombocytopenia (66), though the relationship between thrombocytopenia and increased seizure frequency or additional complications remains unclear. Subsequently, the relationship between GABA transaminase in platelets and GABA transaminase in the brain entered public view (67). Small clinical studies suggested that GABA transaminase levels in platelets of epilepsy patients were relatively higher than in healthy individuals, yet GABA uptake rates were lower than in healthy subjects (68). This phenomenon potentially indicated that antiplatelet therapy for epilepsy could yield potential clinical value, though clinicians at the time interpreted it from the perspective of neural excitation. It wasn’t until 2020 that it was recognized that serotonin released from platelets increases BBB permeability and that platelets can independently stimulate neuronal discharge activity, potentially linked to oxidative stress and neuroinflammation (69). This signifies a shift in the proposed rationale for antiplatelet therapy in epilepsy from neural excitability to neuroinflammation, spanning approximately 40 years.

Platelet-driven amplification of inflammation in the CNS has drawn our particular attention to the biological properties of the calcium-binding protein S100b. S100b is a molecule secreted by astrocytes. Elevated extracellular S100b levels activate TLR7, TLR8, and TLR9 signaling pathways via the adaptor protein TRAF6, leading to activation of nuclear factor κB (NFκB) and mitogen-activated protein kinase (MAPK) (21). This cascade further promotes the release of inflammatory mediators, allowing neuroinflammation to persist. Prolonged or uncontrolled activation of the aforementioned pathways may trap the blood-brain barrier in a vicious cycle of escalating damage. Within this cycle, increased plasma components from the peripheral circulation permeate through the compromised BBB, perpetuating neuroinflammation (20). Without timely intervention, acute neuroinflammation may progressively transition into chronic neuroinflammation.

Clinical evidence suggests that patients with compromised BBB exhibit persistently elevated serum immunoglobulin levels, indicating that antigens from the peripheral circulation have entered the central circulation (20). Experimental evidence supports the notion that BBB disruption triggers neuroinflammation. Immunoglobulin therapy can alleviate neuroinflammation and reduce the extent of neuronal damage. This phenomenon may occur as the BBB gradually repairs itself, with the brain’s lymphatic circulation playing a dominant role in mediating local neuroimmunity (19).

Platelet involvement in neuroinflammation appears to drive neuroinflammation in epilepsy patients by participating in BBB repair, regulating neuroimmune activation, and influencing signaling cascades. This specialized role highlights platelets’ extensive adaptability across peripheral and central immune systems. Platelet-glial-endothelial crosstalk, potentially via the S100B-TLR-NF-κB/MAPK pathway, offers a unified clue for epilepsy-associated neuroinflammation.

**Figure 4:**
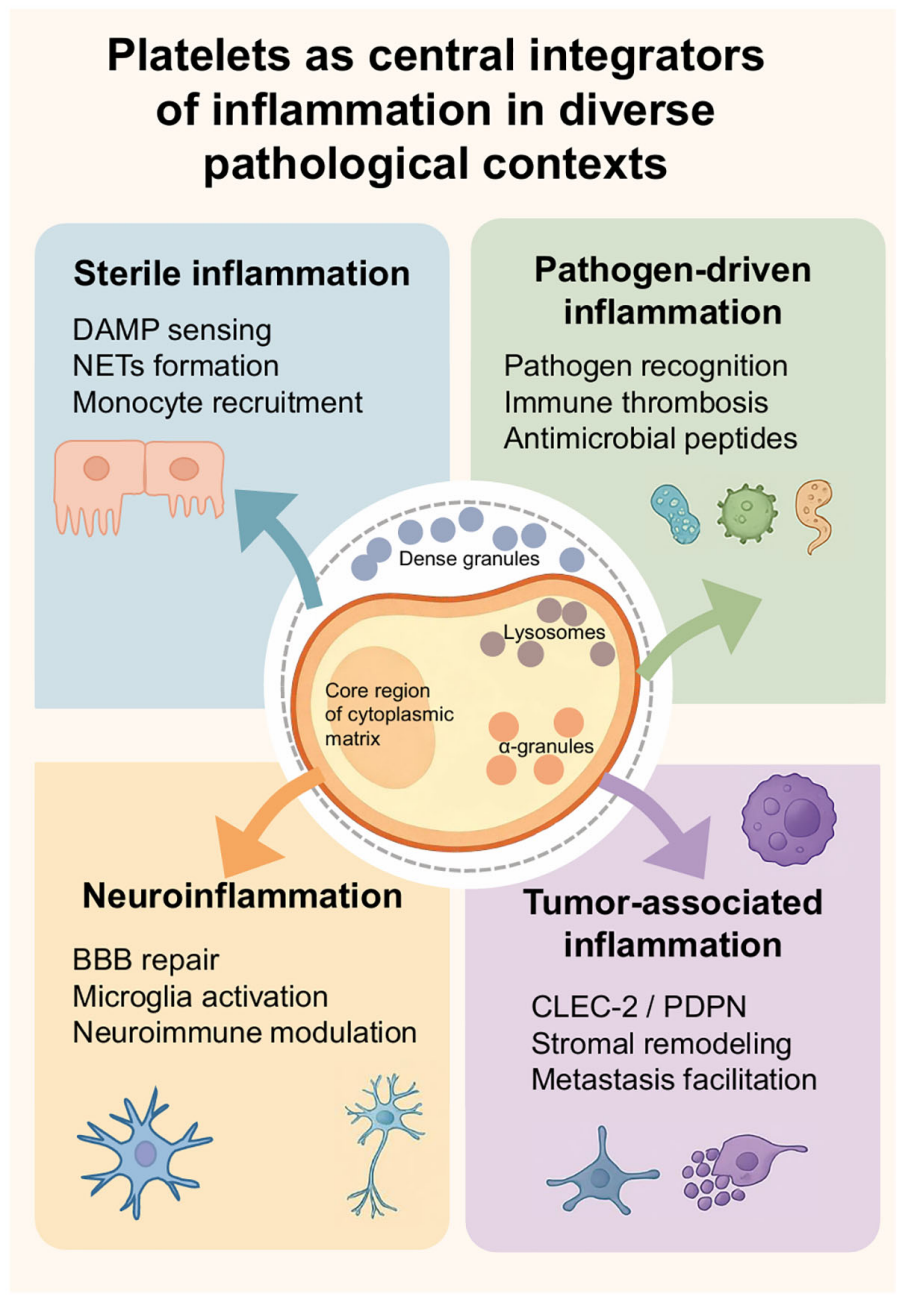
Platelets as central integrators of inflammation in diverse pathological contexts. There are four sections around a central platelet illustration by Figdraw labeled with its internal components: dense granules, lysosomes, and alpha granules. The sections are: Sterile inflammation (DAMP sensing, NETs formation, monocyte recruitment), Pathogen-driven inflammation (Pathogen recognition, immune thrombosis, antimicrobial peptides), Neuroinflammation (BBB repair, microglia activation, neuroimmune modulation), and Tumor-associated inflammation (CLEC-2/PDPN, stromal remodeling, metastasis facilitation). Each section is color-coded with related icons and arrows pointing toward the platelet illustration.

In summary, our observations and evidence-based reasoning indicate that platelets serve as central regulators of inflammatory responses across diverse pathological contexts. The scope of platelet effects depends on the circulatory system and adapts well to specific microenvironments. Whether in aseptic inflammation, pathogen-driven immune activation, tumor inflammation, or neuroinflammation, we observe a central principle of platelet action: regulating the release of specific substances from different platelet granules to mediate injury adhesion, leukocyte recruitment, and the integration of immune and coagulation responses. We attempt to integrate these convergent functions within specific contexts into a conceptual framework that intuitively illustrates the Platelet Inflammation Hypothesis. This positions platelets as a central hub coordinating vascular and immune processes to influence inflammatory outcomes (**Figure 4**). Subsequently, we summarize the clinical significance of the Platelet Inflammation Hypothesis from a translational perspective.

## 5 Clinical implications and therapeutic targeting

Once clinicians recognize platelets as central regulators in immune and inflammatory processes, platelet-targeted therapeutic strategies may yield significant benefits in clinical decision making. Interventions modulating platelet activity can broadly influence the pathophysiology of inflammation, particularly given the dual role of platelet-mediated immune thrombosis in both disease recovery and progression.

Currently, commonly used antiplatelet agents in clinical practice include aspirin or P2Y12 receptor inhibitors. These drugs can mitigate inflammatory responses in specific thrombotic and embolic adverse events. However, such antiplatelet medications are restricted by patient population characteristics; they are currently considered unsuitable for patients at risk of bleeding. This is particularly true when patients remain dependent on platelet function to maintain the host immune environment. Immunologists and pharmaceutical companies are exploring platelet-specific antibodies and receptor antagonists as equivalent alternatives. These may enable targeted modulation of inflammatory pathways in local organs or tissues without interfering with fundamental hemostatic functions. We summarize antiplatelet drugs, their targets, inflammatory effects, and bleeding risks (**Table 1**).

**Table 1:** Candidate interventions targeting platelet-mediated inflammatory programs.

Drug/class	Primary target/pathway	Putative anti-inflammatory or immunothrombotic effects	Bleeding risk (typical signal)
Aspirin (low dose)	COX-1 → ↓TXA_2_	Blunts platelet activation and platelet–leukocyte aggregates; may dampen NETosis and endothelial activation in immunothrombosis	Low–moderate (GI, perioperative)
P2Y_12_ inhibitors (clopidogrel, prasugrel, ticagrelor)	P2Y_12_ (ADP receptor)	Reduces ADP-driven amplification; fewer platelet–neutrophil aggregates; attenuates NET-linked thrombo-inflammation	Moderate–high (agent- and context-dependent; prasugrel/ticagrelor > clopidogrel)
Anti-P-selectin strategies (e.g., crizanlizumab)	P-selectin on platelets/endothelium	Disrupts P-selectin/PSGL-1 adhesion; limits platelet–leukocyte tethering and microvascular immunothrombosis	Low–moderate (bruising/infusion-related; oncology data limited)
PSGL-1 antagonists (investigational)	PSGL-1 on leukocytes/platelets	Similar to above; curtails platelet–leukocyte conjugates and rolling	Unclear (early-phase)
GPIIb/IIIa inhibitors (abciximab, eptifibatide, tirofiban)	Integrin αIIbβ_3_	Potent aggregation block; secondary reduction in thrombo-inflammatory signaling	High (not favored outside PCI or bailout settings)
PAR-1 antagonist (vorapaxar)	Thrombin receptor (PAR-1)	Dampens thrombin-evoked activation; potential reduction in monocyte–platelet complexes	High (intracranial and major bleeding in high-risk groups)
Dipyridamole	PDE inhibition, ↑cAMP; adenosine reuptake block	Anti-adhesive, antioxidant; reports of reduced NET formation and endothelial activation	Low (headache common)
Polyphosphate-targeting approaches (polycationic scavengers, recombinant exopolyphosphatase, enzyme mimetics; investigational)	PolyP-driven contact activation (FXII→FXI)	Blunts NET/PolyP-amplified coagulation without directly impairing primary hemostasis; may curb immunothrombosis	Theoretical low–moderate (clinical evidence pending)
FXII/FXI pathway inhibitors (e.g., garadacimab [FXIIa], abelacimab/milvexian [FXI/FXIa])	Contact pathway proteases	Interrupts NET/PolyP-triggered thrombosis with a hemostasis-sparing profile in early trials	Low–moderate (lower than FXa/IIa anticoagulants; oncology experience evolving)
Heparins (UFH/LMWH)	Antithrombin-mediated anticoagulation; PF4/NET interactions	Reduces thrombin generation; may neutralize histones and NET components; broad anti-inflammatory effects	Moderate (dose- and renal-function dependent)

ADP, adenosine diphosphate; COX-1, cyclooxygenase-1; FX, coagulation factor; LMWH, low-molecular-weight heparin; NET, neutrophil extracellular trap; PCI, percutaneous coronary intervention; PDE, phosphodiesterase; PF4, platelet factor 4 (CXCL4); PolyP, polyphosphate; UFH, unfractionated heparin.

Platelet-directed and contact-pathway interventions with potential to modulate thrombo-inflammatory phenotypes. Entries summarize principal targets, hypothesized anti-inflammatory mechanisms relevant to immunothrombosis, and the prevailing bleeding signal from clinical or early-phase data. See Clinical Implications for narrative synthesis and context-specific considerations.

Potential drug targets for future development can be identified through molecular profiling of specific platelet-derived mediators and surface receptors. For instance, p-selectin or GPIIb/IIIa promote adhesion between platelets and leukocytes; accordingly, inhibitors of these molecules may reduce immune thrombosis and tissue damage dependent on platelet overactivation. Alternatively, antagonists targeting polyphosphate secretion could modulate platelet granule release pathways while attenuating inflammatory signaling without completely inhibiting platelet aggregation function.

The platelet-related indices mentioned earlier can be actively applied in clinical practice. These platelet-related parameters, integrated through mathematical models using biomarkers and routine blood test indicators (platelet count, mean platelet volume, composite inflammatory ratio of platelet values, systemic inflammatory response index), can assess inflammatory responses, infectious immune responses, and tumor disease activity (60). Compared to regions with abundant medical resources, this approach is particularly valuable in resource-poor settings, aiding clinicians in accurately evaluating patient conditions. One reason is that medically underserved areas often lack access to highly precise medical tests, and local patients’ economic circumstances make it difficult to allocate additional disposable income toward such services. This approach not only facilitates personalized treatment strategies after patient stratification but also benefits regions grappling with economic inequality and scarce medical resources.

Currently, Dr. Yan Bo has established the Platelet Inflammation Hypothesis based on literature review and clinical practice reasoning, which lacks secondary validation through clinical or basic research. Furthermore, platelet responses vary significantly across different inflammatory contexts, complicating the design of uniform clinical treatment approaches. How platelets balance protective immunity and pathological inflammation remains a subject for future exploration by immunologists and evidence-based medicine practitioners. Clinicians should focus on integrating real-time monitoring of platelet activity, patient inflammation levels, and clinical symptoms to enhance decision making.

## 6 Future perspectives

Future research into platelet biology is poised to move beyond descriptive associations and toward a more mechanistic and translational understanding of their role in inflammation. A major priority will be to define the context-specific signaling networks that govern platelet activation in different disease settings. This will require integrated approaches that combine molecular profiling, advanced imaging, and functional assays in both experimental models and human subjects.

One promising direction involves dissecting the heterogeneity of platelet responses across various inflammatory states. Single-cell and proteomic technologies may uncover subpopulations of platelets with distinct immunomodulatory profiles, enabling more precise targeting of pathogenic platelet subsets while preserving their protective functions. Such insights could inform the development of therapies that selectively modulate harmful platelet-immune interactions without compromising hemostasis.

The emergence of platelets as potential biomarkers also warrants systematic evaluation. Longitudinal studies that track platelet indices alongside clinical outcomes could clarify their predictive value for disease progression and therapeutic response. The incorporation of platelet-based biomarkers into clinical decision-making algorithms may enable real-time monitoring of inflammatory activity and improve the timing of intervention.

Therapeutically, the challenge will be to translate mechanistic discoveries into safe and effective interventions. This may involve novel drug delivery systems that restrict platelet-targeted agents to sites of inflammation, thereby minimizing systemic effects. Strategies that transiently modulate platelet function during acute inflammatory episodes, rather than continuous suppression, could reduce adverse events associated with long-term platelet inhibition.

Finally, future progress will depend on cross-disciplinary collaboration between immunologists, hematologists, vascular biologists, and clinicians. By integrating expertise from basic science, translational research, and clinical practice, it should be possible to fully exploit the dual role of platelets as sentinels and regulators, ultimately leading to new diagnostic tools and targeted therapies for a broad spectrum of inflammation-driven diseases.

## 7 Conclusion

Platelets serve as the central hub in the microcirculatory-damaged tissue-immune inflammatory interaction network. Their immunoregulatory functions play a pivotal role in diverse inflammatory pathologies. The platelet-driven mechanism-disease-immune inflammatory regulation framework provides clinically translatable insights for diagnostic evaluation of inflammatory and thrombotic conditions, as well as for developing antiplatelet therapeutic strategies targeting diseases such as cancer and epilepsy.
